# A new soft tissue constructed with chitosan for wound dressings-incorporating nanoparticles for medical and nursing therapeutic efficacy

**DOI:** 10.1016/j.reth.2023.06.005

**Published:** 2023-06-17

**Authors:** Minhui Yang, Haibing Wang, Kang Li, Zhongyu Chen, D.T. Seamirumi

**Affiliations:** a Department of Orthopedics, Fuzhou First Hospital of Fujian Medical University, No.190, Dadao Road, Taijiang District, Fuzhou, Fujian, 350000, China; b Department of Pediatric Surgery, Fuzhou Children's Hospital of Fujian Medical University, No.145, No.817 Middle Road, Gulou District, Fuzhou, Fujian, 350004, China; c Department of Orthopedics,The Fifth Hospital of Xiamen, No. 101, Min ‘an Road, Ma Xiang Street, Xiang ‘an District, Xiamen, Fujian, 361101, China; d Faculty of Biomaterials, Advanced Materials Research Center, Isfahan, Iran

**Keywords:** Pediatric hospitals, Hospital-acquired infections, Vital physiological parameters, Innovative wound coverings

## Abstract

The skin, being the largest organ in the human body, plays a vital role in shielding internal organs from external physical and chemical factors. However, skin damage caused by various factors such as injuries, surgeries, diabetes, or burns can lead to wounds that diminish the skin's protective function. Monitoring essential physiological parameters like temperature, moisture, and pH is crucial to facilitate antibiotic treatment, remote physician monitoring, patient comfort, cost reduction, and prevention of hospital-acquired infections. To this end, innovative wound coverings made of biological materials like gelatin, carboxymethyl chitosan, and titanium nanoparticles have been developed, mainly for hospital and pediatric applications. These wound coverings come equipped with sensors to monitor temperature, pH, and moisture and are suitable for pediatric hospitals where children experience difficulty in wound healing due to their sensitive skin. The temperature monitoring feature allows physicians to accurately assess the wound's temperature, detect potential infections, and take prompt actions. These wound coverings can significantly enhance wound treatment for patients, as real-time monitoring of physiological parameters enables informed decision-making by physicians, leading to better therapeutic outcomes. Furthermore, the use of these wound coverings can minimize the risk of hospital-acquired infections. Their adaptability and flexibility make them ideal for various wound types and sizes, ensuring patient comfort and compliance with the treatment plan. In conclusion, the development of innovative and flexible wound coverings using biological materials and equipped with sensors represents a significant breakthrough in wound management. The use of these wound coverings has the potential to revolutionize wound care and improve patient outcomes, particularly in pediatric hospitals where wound healing is often challenging.

## 1 introduction

Wound dressings play a crucial role in wound care, and their effectiveness is paramount in promoting efficient healing. Chitosan-based wound dressings have gained significant interest in recent years due to their biocompatibility, biodegradability, and antibacterial properties. Chitosan is a natural polymer derived from chitin, which is found in crustacean shells and has been extensively studied for its potential in different applications [1]. A wound is a type of injury to the skin caused by physical or chemical factors, including surgery, trauma, burns, or electricity, which may affect the soft tissue, muscle, or bones, and require medical attention. The wound healing process in humans and other evolved animals is highly complex and involves multiple stages, including inflammation, reproduction, repair, and regeneration [2]. Chronic wounds pose significant economic and physiological problems globally, with numerous individuals suffering from them due to accidents or underlying diseases like diabetes [[3], [4], [5], [6]]. Therefore, developing effective treatment strategies that can reduce costs, shorten treatment time, and control wound progression and healing process is crucial [[5], [6], [7], [8]]. Dressings play a critical role in wound healing management, and selecting the appropriate dressing is crucial to successful wound healing by addressing complications and removing the underlying cause. Thus, it is necessary to develop wound dressings that can enhance wound healing and compensate for deficiencies [[9], [10], [11]]. This is particularly important for children, who may have unique requirements for wound dressings, such as flexibility, ease of use, and comfort. Therefore, developing wound dressings that incorporate Iranian gum and antibacterial nanoparticles can significantly improve pediatric wound treatment. These wound dressings can enhance the wound healing process, prevent infections, reduce treatment time and costs, while providing comfort and flexibility to the child. The primary reason for using a wound dressing is to protect the wound from infections and moisture. Despite numerous ointments and commercial products available for wound healing, innovative approaches, including conventional methods such as autologous and allogeneic grafts, have been introduced in wound treatment [[11], [12], [13]].

Wound dressings play a critical role in the management and treatment of various types of injuries [[14], [15], [16], [17], [18], [19]]. Researchers have been exploring different approaches to improve the properties and functionality of wound dressings. Some recent studies have focused on developing 3D fibrous aerogels from polymer nanofibers for use in energy and environmental applications [[20], [21], [22]]. Others have investigated the construction and function of robust and moist bilayer Poly(vinyl alcohol) hydrogels [[23], [24], [25]] and the synthesis and properties of hydrogels with high strength and toughness. Additionally, researchers have explored the use of crosslinked chitosan hydrogels as antibacterial wound dressings [25]. In efforts to develop wound dressings with enhanced properties and functionalities, some researchers have explored the use of materials derived from natural sources. The development of novel wound dressings is a rapidly evolving field with numerous ongoing studies and research efforts [25,26].

Current research focuses on improving wound healing by enhancing the properties and functionalities of wound dressings. The development of advanced wound dressings may improve the quality of life for patients with wounds and injuries, and increase the effectiveness of wound treatment and management. Dressings can be categorized as traditional and modern types, with traditional ones comprising gases such as Vaseline, paraffin, sterile gas, cotton swabs, and bandages, and modern ones consisting of foams, hydrocolloids, hydrogels, alginates, and clear dressings. However, wound dressings are still rudimentary, and none of the current dressings possess all the features of an ideal wound dressing. One of the primary limitations is their inability to provide information about wound healing and environmental conditions, including temperature, humidity, or inflammation. These factors significantly impact the wound healing process, and controlling them is necessary to accelerate healing [27,28].

Smart wound dressings have the potential to offer numerous benefits, such as reducing hospitalization needs, minimizing the risk of infections, lowering physiological injuries, and decreasing treatment costs for patients. In recent years, there has been considerable interest in tissue engineering for repairing and replacing damaged tissues. Incorporating electronic sensors, such as those measuring pH, temperature, humidity, and oxygen, into smart wound dressings can monitor wound conditions and potentially control wound infections. Intelligent wound dressing systems that can manage wound environments without hospitalization represent one of the most innovative biomedical advances in wound diagnosis and treatment [[29], [30], [31], [32], [33]]. The objective of this article is to develop a smart wound dressing by combining tissue engineering and bio-electrics to expedite the wound healing process using natural wound tissue [34]. Smart wound dressings have the potential to reduce costs for patients and physicians by eliminating risk factors and selecting the most effective and cost-efficient treatments, particularly for pediatric applications.

## 2 materials and methods

### 2.1 Preparation of novel wound dress

Carboxymethyl Chitosan (CMC) and gelatin are popularly used in wound dressings due to their non-toxic properties, high adsorption power, availability, degradability, environmental compatibility, and cost-effectiveness. The combination of CMC and gelatin with titanium dioxide nanoparticles (TiO_2_) can activate coagulation pathways, making the wound dressing materials antibacterial and compatible with the body. For this study, wound dressing materials were prepared using polymers and ceramic materials of 98% purity from Merck company, Germany. A mixture of CMC, gelatin, and distilled water was stirred on a magnetic stirrer and hot plate at 55°C and 450 rpm. The resulting solution was evaluated for its acidity, which was found to be in the optimal range of neutral to slightly acidic for wound dressing applications. During this experiment, a laboratory pH meter was calibrated using buffer solutions of pH 4 and pH 7. The pH of hypochlorite and lemon juice was then measured, indicating an alkaline pH of 12 for hypochlorite and an acidic pH of 2 for lemon juice. A base material was chosen, and distilled water was added to create materials spanning pH values from 2 to 12. TiO_2_ nanoparticles were added to the resulting solutions, which were then heated on a hot plate for 30 min at 40 °C and 200 rpm. A portion of the solution was transferred to a Petri dish and frozen at −50 °C for 24 h, followed by freeze-drying at −55 °C for another 24 h. Biological wound dressings, constructed from biological materials, are utilized for children with a variety of wound types. These dressings are produced using cells, bacteria, or other biological materials, and have the ability to regulate the wound environment. Biological wound dressings offer several advantages for pediatric applications, including a reduced risk of local infections, accelerated wound healing rates, and alleviation of pain and inflammation symptoms. Additionally, they are safe from harmful side effects or chemical components that may pose a threat to children. Biological wound dressings provide an efficient and secure solution for various types of wounds in children. In recent times, biological wound dressings have gained popularity due to their numerous advantages over conventional wound dressings. Biological wound dressings are made from natural, biological materials such as collagen, hyaluronic acid, and chitosan, which have the ability to stimulate wound healing and are biocompatible with human tissue. These dressings are particularly advantageous for pediatric patients as they have a lower probability of causing adverse reactions or side effects. Furthermore, these dressings can be customized to meet the unique needs of each patient by regulating the wound environment, controlling moisture levels and oxygen supply, and providing antimicrobial properties to prevent infection.

### 2.2 Analysis of the wound dress

#### 2.2.1 XRD analysis

To produce and evaluate advanced wound products, the X-ray Diffraction (XRD, Philips, X pert, Research Materials Center) device was employed to examine the crystal structure of chitosan. The analysis confirmed that the chitosan peak index is found in the range of 15°–25°, with a small number of titanium nanoparticles leading to an increase in intensity. Chitosan, a natural amino polysaccharide, has garnered significant attention from various industries, such as textile, medicine, tissue engineering, and wound healing, due to its unique structure, multidimensional properties, and exceptional performance. The use of polymers in medical settings, particularly in wound dressings, membranes, and cosmetics, has become a significant area of focus. Additionally, the chemical modification of these biopolymers has improved their solubility in aqueous media, thereby enhancing their biological activity and mechanical features.

#### 2.2.2 SEM analysis

In this study, the morphology and porosity of a wound dressing were analyzed using Scanning Electron Microscopy (SEM) on a Zeiss LEO 1530-1 FESEM/EDS/EBSD. SEM is a powerful tool used to investigate the structure and surface of materials with high resolution. The purpose of this analysis was to investigate the wound dressing's structure and evaluate its suitability for promoting wound healing. The SEM images revealed that the wound dressing's cavities have a homogeneous porosity, indicating that the dressing's structure is suitable for promoting wound healing. The interconnected nature of the cavities is also a desirable feature, as it allows for the efficient distribution of fluids and gases, promoting a favorable healing environment. The observed pore size range of 60–90 nm is also optimal for supporting cell proliferation and migration, which are critical processes for wound healing.

### 2.3 Evaluation of soft wound dressing

Mechanical strength is a crucial property of wound dressings as it must be able to withstand various stresses. To evaluate the tensile strength (TS) and elastic modulus of the wound model, the tensile strength test was conducted following the American Society for Testing and Materials (ASTM-ST50) standard. This study aimed to evaluate the TS results of bio-nanocomposite wound dressings containing biopolymer and bioceramic, enhancing their use in smart wound dressings. Statistical analysis of the TS results using SPSS software showed a P-value of 0.92 for the SHT20 sensor and 0.05 for the digital hygrometer (Table 1 and Table 2). Since the sample size was less than 50, the Shapiro Wilk test was utilized. To ensure the performance of the color detection sensor, the sensor was first placed in front of the red color spectrum to verify its sensitivity to various acidic and alkaline substances and the color detection power of the microcapsules. Subsequently, the microcapsules were changed in the presence of different alkaline and acidic substances, and their color changes in terms of the red color frequency were recorded. The pH of the wound was determined, and a microcontroller program was designed based on these changes.

**Table 1:** Report the results of humidity test by SHT20 sensor and digital hygrometer.

Repeat the Experiment	Humidity read by SHT20 sensor	Humidity read by Digital Hygrometer
**1**	28.20	28.10
**2**	28.80	28.70
**3**	31.00	30.90
**4**	30.50	30.40
**5**	29.70	29.50
**6**	28.90	28.70
**7**	34.00	33.00
**8**	32.00	32.80
**9**	28.20	24.10
**10**	29.40	28.30
**11**	30.20	30.00
**12**	30.30	32.00
**13**	33.00	32.50
**14**	31.50	31.00
**15**	29.80	29.50
**16**	28.50	28.70
**17**	29.10	29.50
**18**	26.20	26.10
**19**	30.50	30.40
**20**	25.60	25.70

**Table 2:** Report the results of temperature test by digital thermometer and SHT20 sensor in terms.

Repeat the experiment	Temperature read by SHT20 sensor (°C)	Temperature read by the digital thermometer (°C)
**1**	34.6	35.3
**2**	35.2	35.4
**3**	35.4	35.4
**4**	35.4	35.5
**5**	35.5	35.6
**6**	35.7	35.7
**7**	35.8	35.8
**8**	36.1	36.1
**9**	36.1	36.2
**10**	36.4	37.2
**11**	37.6	37.5
**12**	38.6	38.8
**13**	40.1	40.0
**14**	40.1	40.3
**15**	41.4	41.5
**16**	42.2	42.3
**17**	42.6	42.8
**18**	43.2	43.1
**19**	43.5	43.4
**20**	43.7	43.7

To evaluate the accuracy of the SHT20 sensor, a digital hygrometer was used to measure the humidity in different areas simultaneously. This experiment was repeated in 20 different locations, and the results were statistically analyzed using the Shapiro Wilk test because the sample size was less than 50. The Pearson correlation test was employed to examine the relationship between the color and pH value variables, which were found to have a normal distribution. The results of the Pearson test indicated a correlation coefficient of 0.63 and a p-value of 0.003 for these two variables. Since the p-value was less than 0.05, it can be concluded that this relationship is significant and can be generalized to the entire population. In this study, we propose a new soft tissue construct for wound dressings using chitosan, incorporating nanoparticles to enhance medical and nursing therapeutic efficacy. The incorporation of nanoparticles into wound dressings has been shown to improve their mechanical properties, antibacterial activity, and drug delivery capabilities. We aim to investigate the potential of our chitosan-based wound dressing with incorporated nanoparticles to improve wound healing outcomes. This study aims to analyze the morphology and porosity of the wound dressing using scanning electron microscopy (SEM) and evaluate its mechanical strength using tensile strength testing. Furthermore, we will investigate the wound dressing's ability to regulate the wound environment by controlling moisture levels and oxygen supply, providing antimicrobial properties to prevent infection.

## 3 Results and discussion

**Fig. 1:**
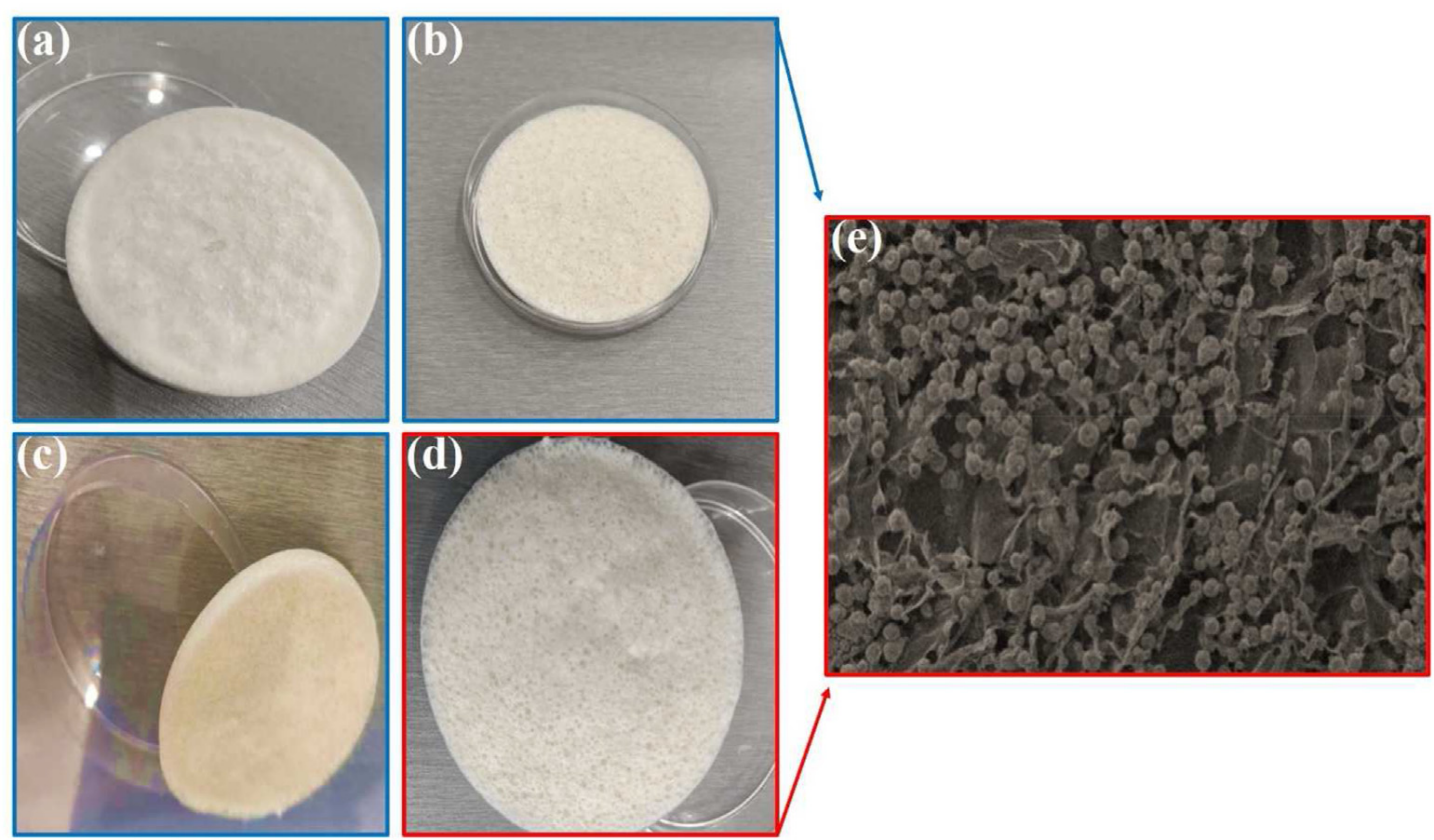
a) Image of the top surface, b) top surface of the sample in the Petri dish, c) thickness of the sample, d) porosity of the sample surface and f) microscopic image of the wound dressing.

**Fig. 2:**
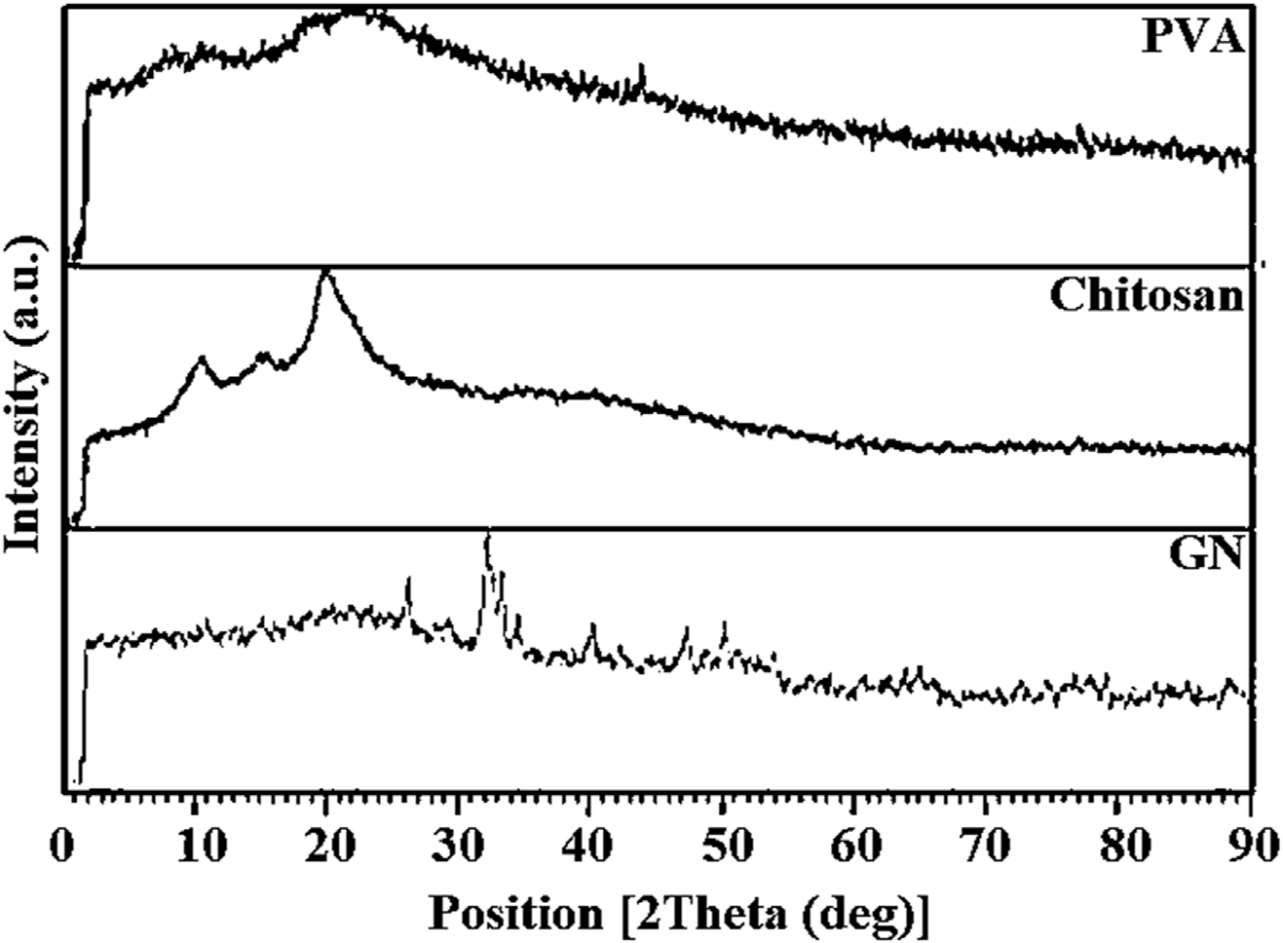
2 D, XRD pattern of the base polymers of polyvinyl alcohol, chitosan and gelatin to prepare the novel wound dress.

**Fig. 3:**
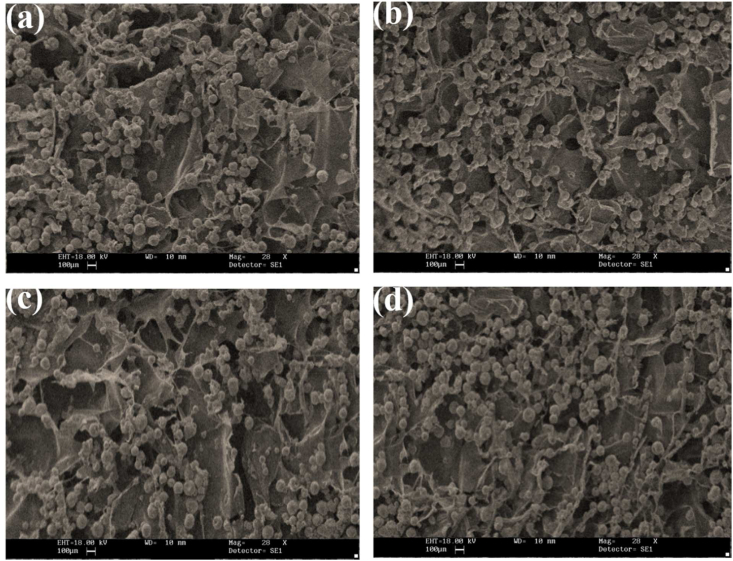
SEM images of a sample containing various amounts of chitosan with addition of a) 0, b) 2.5, c) 5, and d) 7.5 wt% for wound healing application before soaking in PBS saline.

Fig. 1 includes a series of images and measurements that allow for a thorough understanding of the examined wound dressing sample. Panel a) displays the image of the sample's top surface, which enables observation of the texture and morphology of the material. Panel b) showcases the top surface of the sample in the Petri dish, providing additional information on its size and shape. Panel c) presents the thickness of the sample, a critical parameter in determining its mechanical properties such as hardness and elasticity. Thickness measurements can also assist in optimizing the amount of wound dressing material required to cover a specific wound size. Panel d) indicates the porosity of the sample surface, a key factor in the wound healing process's effectiveness. The presence of pores in the wound cover promotes better air circulation, gas exchange, and nutrient flow between the wound tissue and the environment, thereby accelerating the wound healing process. Finally, panel e) reveals the microscopic image of the wound dressing sample, offering a detailed view of its internal structure and aiding in the understanding of its properties and behavior. By analyzing the microscopic images, researchers can gain insights into wound dressing composition, including the type and distribution of materials used, and the material's ability to interact with the wound bed and promote healing. The various measurements and images presented in Fig. 1 provide a comprehensive view of the wound coverage pattern, which is crucial in optimizing wound management and improving patient outcomes. Fig. 2 displayed the XRD pattern, confirming the base polymers of Polyvinyl Alcohol (PVA), chitosan (CH), and Gelatin (GN) that were used in the production of the new wound dressing. The results of the analysis reveal that the wound dressing possesses the necessary morphological and porosity characteristics that are conducive to the process of wound healing. These results are significant in terms of advancing the development of better wound dressings that can accelerate the healing process and improve the outcomes for patients. Further studies are required to determine the efficacy of this wound dressing in clinical settings. XRD analysis was performed to determine the morphology of bio-nanocomposites and confirm the presence of titanium oxide nanoparticles in the prepared flexible bio-nanocomposites. The XRD pattern showed sharp peaks at an angle of 2 θ = 22.7 and two peaks at angles of 16° and 7.4° for the gelatin. The crystal structure and dimensions of the PVA and gelatin lattice unit were accurately matched, indicating their high similarity. Morphology and structure of the nanoparticles and the polymer reaction in four wound dressing samples are analyzed by means of SEM, and presented in Fig. 3(a–d). The distribution and dispersion of nanoparticles were found to be inhomogeneous in bio-nanocomposite samples with weight percentages of 0, 2.5, 5, and 7.5 titanium nanoparticles, resulting in varying degrees of porosity. After conducting microanalysis, it was discovered that the titanium nanoparticles had a size range of 10–30 μm, and exhibited a strong adhesion to the chitosan polymer. The amount of titanium nanoparticles used in the production process directly impacted the porosity and morphology of the wound dressing. Fig. 3(b) shows the spherical-shaped TiO_2_ nanoparticles were observed in the sample. By increasing the additive up to 7.5 wt%, the porosity became homogeneous and continuous with a size range of 100–200 nm, as shown in Fig. 3(c). However, the scattering of particles was uneven, as illustrated in Fig. 3(d) before soaking in the phosphate-buffered saline (PBS). The SEM images also show that the addition of chitosan to the wound dressing sample promotes the formation of a porous structure. The size and distribution of the pores in the sample vary with the amount of chitosan added. The porous structure is crucial for facilitating air and fluid flow, promoting oxygen and nutrient exchange, and accelerating wound healing.

**Fig. 4:**
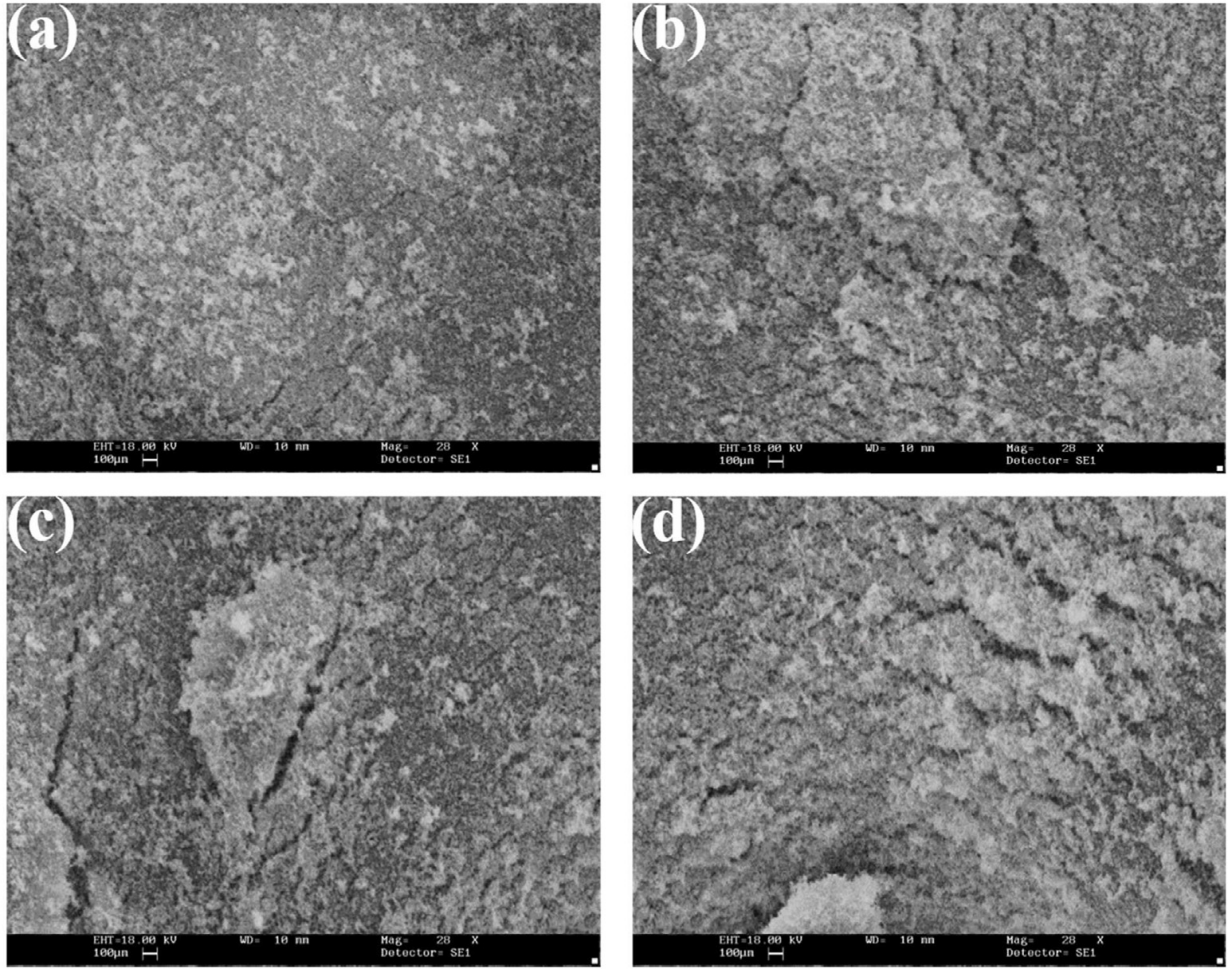
SEM images of a sample containing various amounts of chitosan with addition of a) 0, b) 2.5, c) 5, and d) 7.5 wt% of TiO_2_ for wound healing application after soaking in PBS saline.

**Fig. 5:**
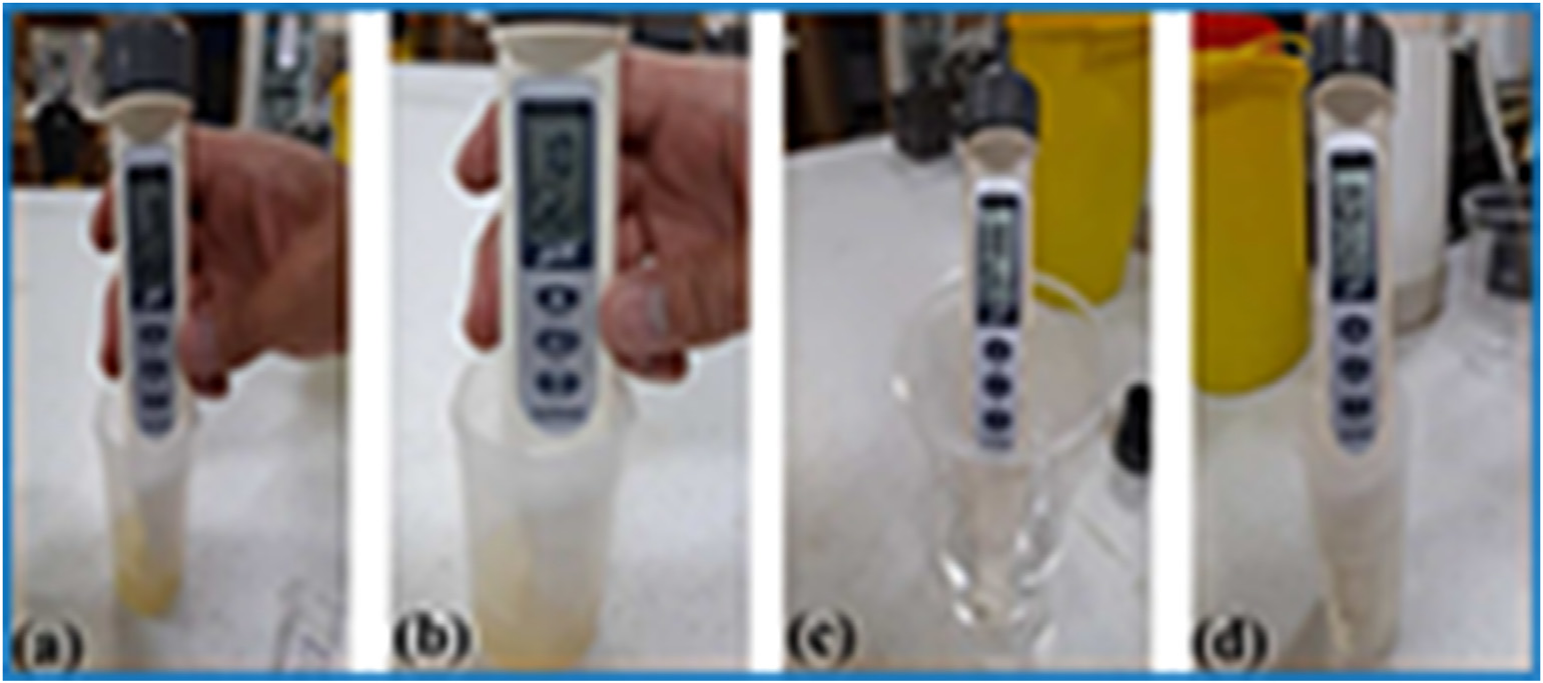
The concentration of the samples in phosphate buffered saline solution was checked after 48 h, and the findings were recorded after 48 h with addition of a) 0, b) 2.5, c) 5, and d) 7.5 wt% types of samples.

The SEM images provide a detailed view of the sample's surface morphology and microstructure after exposure to PBS saline. The images show that the porous structure observed in the SEM images in Fig. 4 is still present, indicating that the wound dressing sample has maintained its structural integrity after exposure to PBS saline. The SEM images also reveal that the addition of TiO_2_ to the wound dressing sample alters its surface morphology after exposure to PBS saline. The images show the formation of structures such as pores, ridges, and bumps, which are similar to those observed in the SEM images in Fig. 4. However, the size and shape of these structures appear to be different, indicating that exposure to PBS saline has affected the material's surface morphology. The SEM images provide information on the material's stability and durability after exposure to PBS saline. The preservation of the porous structure is crucial for facilitating air and fluid flow and promoting oxygen and nutrient exchange, which are essential for the wound healing process. The formation of new structures on the surface of the sample after exposure to PBS saline provides insights into the structure and potential degradation. This knowledge is useful in understanding the material's behavior in a real-world wound healing application and optimizing the manufacturing process to improve its properties. Furthermore, the SEM images in Fig. 4 provide a comparison to the SEM images in Fig. 4, which were captured before exposure to PBS saline. By comparing the images, researchers can gain insights into the material's changes after exposure to a physiological environment. This information can be used to optimize the manufacturing process and improve the wound dressing's properties, stability, and durability. Fig. 5 shows the concentration of the samples in a PBS was measured after 48 h, and the findings were recorded. PBS is a commonly used solution that mimics the body's internal environment and is used to evaluate the biocompatibility of materials. The concentration measurement is an essential parameter to determine the material's stability and durability in PBS solution. The measurement is typically performed using a spectrophotometer or other analytical instruments, which can detect changes in the sample's optical properties resulting from the interaction with PBS solution. The measurement of concentration after 48 h provides critical information on the material's behavior in a physiological environment. The concentration change can indicate the material's degradation rate and potential impact on wound healing efficacy. A significant concentration change may suggest that the material is not stable or durable in the physiological environment, which can affect its efficacy in a wound healing application. The concentration measurement can be used to optimize the manufacturing process and improve the material's properties for better wound healing outcomes. By analyzing the concentration data, researchers can identify the optimal amount of the material to use in a wound dressing to ensure its stability and durability in a physiological environment.

**Fig. 6:**
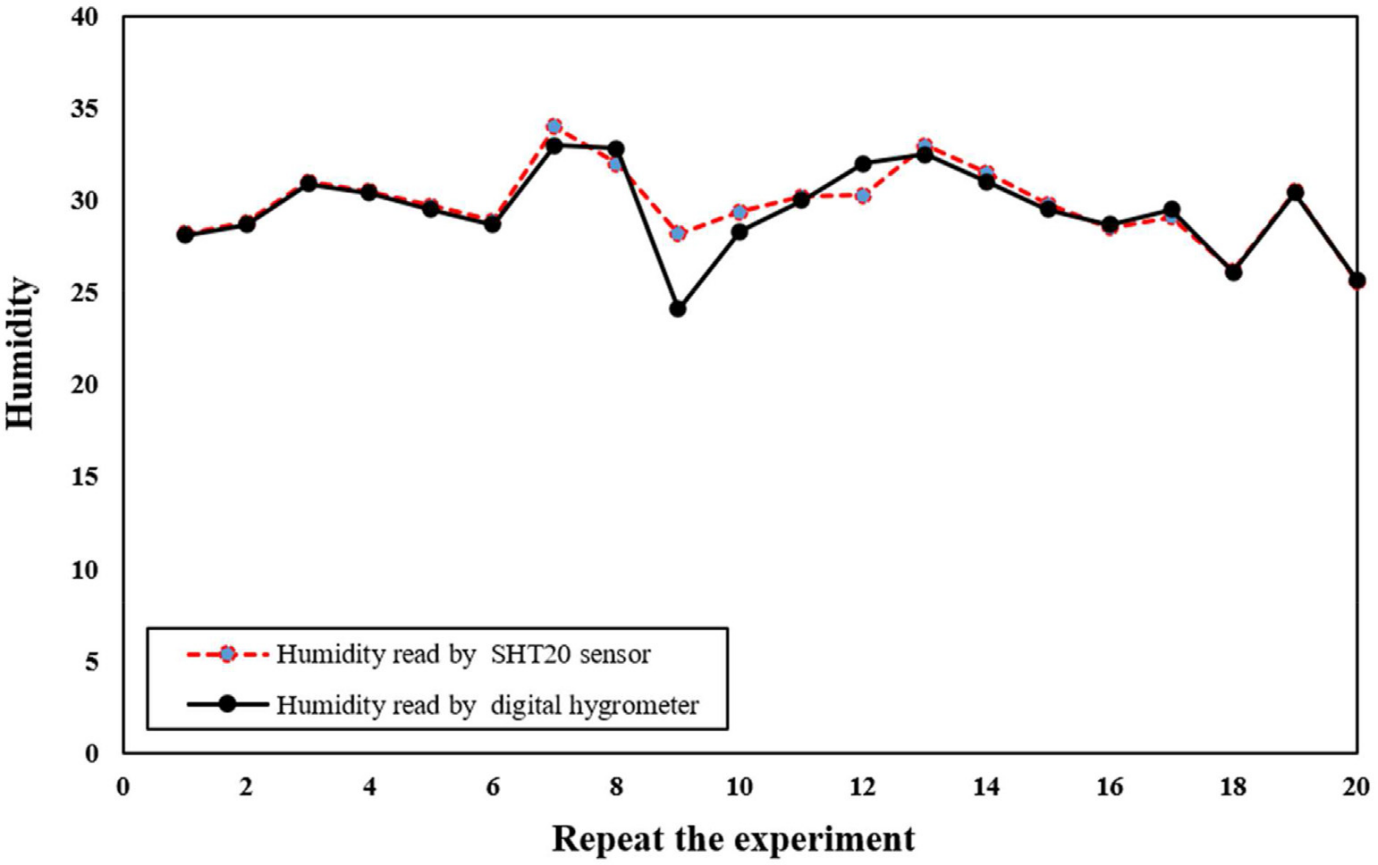
The use of a digital hygrometer and a sensor enabled us to monitor the sample's moisture level after a certain time.

**Fig. 7:**
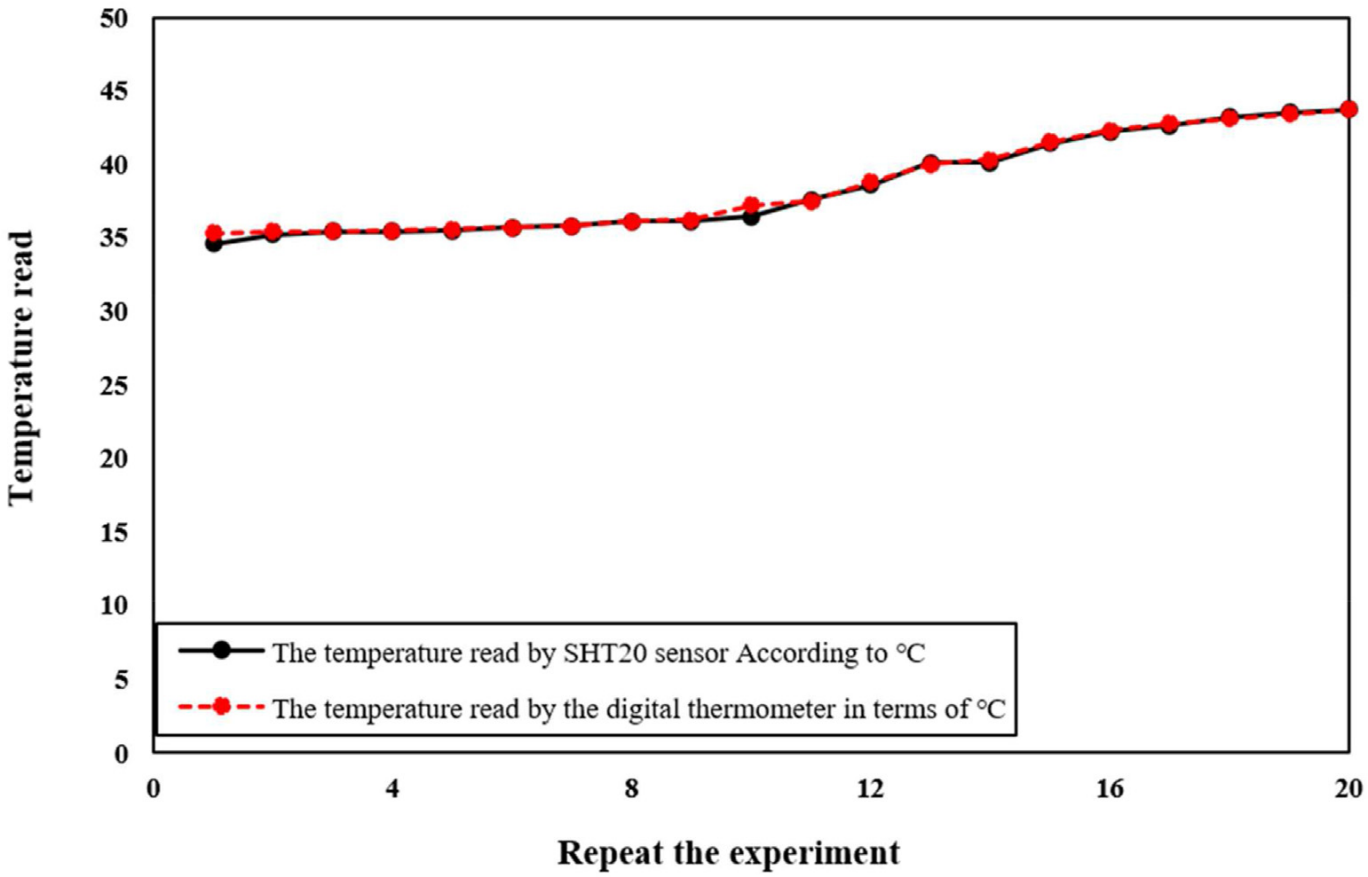
The use of a digital temperature enabled us to monitor the sample's temperature level after a certain time.

Fig. 6 illustrates the use of a digital hygrometer and a sensor to monitor the moisture level of a wound dressing sample over a period of time. Moisture level is a crucial parameter in wound healing because it affects the wound dressing's ability to maintain an optimal moist environment that supports cell migration, proliferation, and differentiation. To measure the moisture level of the sample, the digital hygrometer and sensor were placed in the sample's environment, providing real-time data on the relative humidity and temperature. The collected data enables researchers to evaluate the material's ability to maintain a moist environment and optimize the manufacturing process to improve the material's properties for better wound healing outcomes. The use of the digital hygrometer and sensor is an essential tool in wound healing applications, as it provides valuable insights into the material's behavior under different environmental conditions. Fig. 7 demonstrates the use of a digital temperature sensor to monitor the temperature level of a wound dressing sample over a period of time. Temperature is a critical parameter that can affect the wound dressing's performance, stability, and biocompatibility. The digital temperature sensor is a commonly used instrument that measures the sample's temperature in real-time. By placing the sensor in the sample's environment, researchers can detect changes in temperature resulting from the interaction with the surrounding environment. The temperature data obtained from the sensor can provide valuable insights into the material's behavior under different temperature conditions, allowing researchers to optimize the material's properties for better wound-healing outcomes. Monitoring the sample's temperature level is crucial in wound healing applications as a change in temperature can impact the material's stability, durability, and biocompatibility, which can affect its efficacy in wound healing. Therefore, the use of a digital temperature sensor is an important tool in wound healing research, providing researchers with real-time data to evaluate the material's behavior under different temperature conditions and optimize the manufacturing process to improve the material's properties for better therapeutic outcomes. In order to detect color changes in the wound dress after implantation, a reference color is employed to compensate for variations in colorimetric data obtained from the microfibers in each image. By utilizing multiple imaging modalities, including RGB, more precise pH measurements can be obtained. Notably, the process of extracting RGB values for different individuals has been calibrated for varying background changes and light conditions. The accuracy of the sensors was evaluated using the SHT20 sensor, which was repeated over 20 times. Fig. 7 shows the relationship between the SHT20 sensor and the digital thermometer. To measure temperature, humidity, and pH, an AVR microcontroller with sensors was embedded in the wound dress. An alarm interval was set for these parameters, and warning data can be transmitted to a doctor via SMS. The doctor can also request all the parameters to be sent to a specialist daily. In this research, a sensor thermometer and hygrometer were utilized to detect temperature and humidity changes. The thermometer is capable of detecting changes in temperature as small as 0.01°C (0.04° in 12-bit mode) with a resolution of 14 bits (variable to 12 bits). However, the average error in temperature detection is 0.3°C. The hygrometer can detect a 0.04% increase or decrease in humidity levels of 0.7% in 8-bit mode with a resolution of 12 bits (variable to 8 bits). Nevertheless, the average error in moisture detection accuracy is 3%. Since pH sensors can only function in immersed solutions, the TCS3200 color recognition module was utilized to measure this parameter. The module works by converting colors to corresponding frequencies and sending digital signals to a microcontroller. It has high resolution, color output adjustment, and an error rate of 0.2% at 50 kHz frequency. Moreover, it can detect an unlimited range of colors. The study also employed microcapsules with a diameter of 100–200 μm, which were sensitive to pH and produced using an electrospray device. These microcapsules were placed inside a wound to measure pH levels. Additionally, the module can be utilized to read, test, calibrate, arrange, and match different colors. In this study, we investigated the appropriateness of commercially available elements for wound dressings and found that their dimensions make them unsuitable. We also discovered that the SIM800C module used in our system experiences a 10-s delay during program initiation. To activate various components, including relays, SHT20 sensors for temperature and humidity measurement, and pH sensors, which communicate through the microcontroller's two-wire protocol, we utilized Port A of the microcontroller, as shown in Fig. 7. To facilitate the wound healing process, we developed an intelligent and flexible wound dressing equipped with temperature and pH sensors that can continuously monitor wound conditions. Using the SIM800C module, we programmed the microcontroller to send SMS alerts to physicians when the target parameters exceed allowable limits. Wound dressings are a critical aspect of wound care, and various approaches have been developed to improve their efficacy and accelerate the healing process. In oncology patients, commercially available honeys have been investigated as a potential treatment for oral mucositis [35]. Essential oils of pennyroyal (Mentha pulegium L.) have also been found to have great health benefits and may be a natural and organic medicine for wound healing [36]. Microfluidic electrospray niacin metal-organic frameworks encapsulated microcapsules have been developed for wound healing [37], and a composite indicator dressing has been shown to enable continuous pH monitoring in wounds, demonstrating its feasibility as a potential solution for wound care [38]. These studies highlight the potential of various approaches to improve the efficacy of wound dressings and promote wound healing. We anticipate that our new soft tissue construct with chitosan and nanoparticles will demonstrate improved therapeutic efficacy in wound healing compared to existing wound dressings. Our findings have the potential to contribute to the development of more effective and efficient wound dressings, which could significantly improve patient outcomes and reduce healthcare costs.

## 4 conclusion

The study has shown that the wound dressing possesses desirable mechanical and biological properties, such as appropriate elasticity, oxygen permeability, and pH sensitivity. The dressing incorporates microcapsules that contain sensors for humidity, temperature, and pH levels. The p-values being less than 0.05 indicate that the results are statistically significant and can be generalized to the entire population. Based on these findings, it can be concluded that the SHT20 sensor is suitable for research purposes. The use of chitosan-based wound dressings also has significant advantages over conventional wound dressings. Chitosan's biodegradability and biocompatibility make it a safe and sustainable option, reducing the risk of allergic reactions and other adverse events. Furthermore, chitosan's antibacterial properties can help prevent infection, which is crucial in wound healing. The results indicated that the SHT20 sensor is a viable alternative for measuring humidity in different environments. The TCS3200 sensor readings showed that the red frequency frequencies ranged between 98 and 198, corresponding to the contact of the materials with the skin of the hand and wound location on the skin. Through this study, we aim to contribute to the development of more effective wound dressings that can improve patient outcomes and reduce healthcare costs. We believe that the incorporation of nanoparticles into chitosan-based wound dressings could be a promising approach and could pave the way for the development of innovative wound care solutions.

## Declaration of competing interest

The authors declare that they have no known competing financial interests or personal relationships that could have appeared to influence the work reported in this paper.
